# Staphylococcus warneri: Skin Commensal and a Rare Cause of Urinary Tract Infection

**DOI:** 10.7759/cureus.8337

**Published:** 2020-05-28

**Authors:** Aparna Kanuparthy, Tejo Challa, Sreenath Meegada, Suman Siddamreddy, Vijayadershan Muppidi

**Affiliations:** 1 Internal Medicine, University of Texas Health Science Center/Christus Good Shepherd Medical Center, Longview, USA; 2 Internal Medicine, Baptist Health Medical Center, Little Rock, USA; 3 Internal Medicine, Indiana University Health, Indianapolis, USA

**Keywords:** staphylococcus warneri, uti, skin commensal, liver disease

## Abstract

Coagulase negative Staphylococci often grow in cultures and form one of the most abundant flora among skin microbiome. It is important and challenging to identify and treat clinically significant infections caused by these organisms. Prosthetic devices, catheters and conditions causing immunocompromised states are the risk factors for such infections. We describe a case of clinically significant and symptomatic urinary tract infection (UTI) in a 65-year-old man with liver cirrhosis caused by Staphylococcus warneri which forms <1% of Staphylococcal skin flora. He was treated successfully with fluoroquinolone antibiotic based on culture results. It is important to understand potential of this organism to cause serious infections which warrant culture-directed antibiotic therapy.

## Introduction

Coagulase-negative staphylococci are skin commensals [1-4]. They are often considered as contaminants in clinical specimens. These organisms present a clinical challenge in differentiating contamination from clinically significant infection when they grow in cultures. There has been increased recognition of the clinical relevance of the presence of these organisms in cultures. The risk factors for clinically significant infections with these organisms include immunosuppression, prosthetic devices, and intravascular catheters. Staphylococcus saprophyticus has been recognized as the most common cause of coagulase-negative urinary tract infection (UTI). Staphylococcus warneri is a rare cause of UTI [3]. We describe the case of Staphylococcus warneri UTI in patient with liver cirrhosis resistant to beta-lactam antibiotics. Liver cirrhosis, irrespective of the cause, is known to cause immunocompromised state [5]. Cirrhosis impairs several aspects of immune response related to both innate and adaptive immunity [6,7].

## Case presentation

The patient is a 65-year-old male with a history of cirrhosis of liver due to alcohol abuse, atrial fibrillation, hypertension, obstructive sleep apnea, hyperlipidemia, and history of deep venous thrombosis (DVT). He presented to the hospital with generalized weakness, pain with urination, and urinary frequency with a need to void every 15 minutes. This was associated with episodes of incontinence. The patient did not report any fevers or chills, had nausea, vomiting. He had intermittent episodes of a small amount of blood in the stool. He continues to drink heavily and has a history of smoking. The patient was intoxicated with alcohol at presentation. He did not have any history of urological problems or a history of previous urinary tract infections requiring treatment. He has no significant family history of liver disease or immunological disorders.

On physical examination, the patient was afebrile with normal blood pressure, pulse, and oxygen saturation. Heart and lung sounds were normal; the examination of the abdomen was significant only for mild epigastric tenderness, no significant costo-vertebral angle tenderness. The patient was euvolemic without significant ascites, peripheral edema. The patient had pancytopenia, urine toxicology screen was positive for alcohol, and cannabis, and urinalysis showed amber-colored urine with leukocytes, positive leukocyte esterase, and negative nitrite (Tables 1, 2).

**Table 1:** Complete blood cell count showing pancytopenia secondary to chronic liver disease

White blood count	3200/mm^3^
Red blood count	3.44 million/mm^3^
Hemoglobin	11 gram/dL
Hematocrit	31.8%
Platelet count	39,000/mm^3^
Segmented neutrophils	77%
Band neutrophils	1%
Lymphocytes	19%

**Table 2:** Urine analysis showing elevated white blood cell count, positive leukocyte esterase

Urine color	Amber
Urine pH	6.0
Urine protein	30 mg/dL
Urine blood	Moderate
Urine bilirubin	Small
Urine leukocyte esterase	Positive
Urine nitrite	Negative
Urine red blood cell count	3 cells/high power field
Urine white blood cell count	161 cells/high power field

The patient was admitted to the hospital, monitored for alcohol withdrawal, gastro-intestinal bleeding, and was started on empiric treatment with ceftriaxone for urinary tract infection. There was no recurrence of rectal bleed during the hospital course, and the patient did require benzodiazepines for alcohol withdrawal. The patient’s MELD score was 17. Urine cultures were sent. Cultures grew coagulase-negative staphylococci. Subsequently, the organism was identified as Staphylococcus warneri with 75,000-100,000 colony forming units/ml on culture results. The organism is resistant to Penicillins and was beta-lactamase producing (Table 3).

**Table 3:** Urine culture showing organism and sensitivities

Staphylococcus warneri	75,000-100,000 colony forming units/ml
Sensitivities:	
Ciprofloxacin	Sensitive
Daptomycin	Sensitive
Gentamycin	Sensitive
Levofloxacin	Sensitive
Linezolid	Sensitive
Nitrofurantoin	Sensitive
Oxacillin	Sensitive
Penicillin	Resistant
Rifampin	Sensitive
Trimethoprim/sulfamethoxazole	Sensitive

It was sensitive to fluoroquinolone antibiotics and Bactrim. The patient was started on levofloxacin with improvement. The patient did have an episode of bleeding from the penis, which required a urology evaluation. Urologist recommended culture-directed antibiotics and possible cystoscopy if there is a recurrence of hematuria. The patient’s dysuria and urinary frequency resolved. There was no hematuria or recurrence of bleeding from the penis. The patient was discharged home on oral levofloxacin with scheduled outpatient follow-up. The patient was encouraged to quit drinking alcohol. He was given alcohol cessation counseling and resources for alcohol cessation.

## Discussion

It is important to recognize the role of coagulase-negative Staphylococci in causing UTIs, among other serious infections. Their role in the pathogenesis of such infections has drawn more attention in the past several years. Staphylococcus saprophyticus, S. epidermidis, S. haemolyticus and S. warneri account for most of the responsible organisms [8]. S. saprophyticus is the most common cause of UTIs. S. warneri is catalase positive, oxidase negative, coagulase-negative organism, which is a commensal on skin flora. It constitutes <1% of skin Staphylococcus flora [9]. It is a rare cause of sepsis, and an immunocompromised state is a predisposing factor for such infections [10]. There are reported cases of catheter-related bacteremia, endocarditis, multiple abscesses, septic arthritis caused by Staphylococcus warneri [10,11]. Isolates from these infections are generally resistant to Penicillins, with beta-lactamase activity and show susceptibility to other antibiotics with gram-positive activity. Infections due to these organisms were successfully treated with fluoroquinolones, among other antibiotics [12].

## Conclusions

Coagulase-negative Staphylococci are frequently isolated from urinary specimens. Absence of symptoms, pyuria, and the presence of other skin and urethral flora help exclude clinically significant infections. Urine samples, in many cases, may show growth of <100,000 colony forming units (CFU). Appropriate setting based on clinical symptoms, presence of other commensal skin, and urethral organisms in urine help determine the clinical significance of the culture results. Staphylococcus warneri, which constitutes a tiny part of skin flora, may cause significant infections. Based on the clinical picture and above discussed criteria, clinically significant infections should be treated based on culture results. Non-beta-lactam antibiotics with gram positive activity can successfully treat these infections based on the data available so far.
